# Development and evaluation of a synthetic seed system for cassava propagation

**DOI:** 10.1371/journal.pone.0356235

**Published:** 2026-08-14

**Authors:** Leila Verena da Conceição, Diego Fernando Marmolejo Cortes, Marcos de Souza Campos, Lucas Lenin Resende de Assis, Alessandro Pereira Gomes de Jesus, Fernanda de Azevedo Souza, Eder Jorge de Oliveira

**Affiliations:** 1 Universidade Federal do Recôncavo da Bahia, Campus Cruz das Almas, Cruz das Almas, Bahia, Brazil; 2 Embrapa Mandioca e Fruticultura, Cruz das Almas, Bahia, Brazil; Prince of Songkla University, THAILAND

## Abstract

Cassava is a crop of major global importance for food security; however, its conventional vegetative propagation through stem cuttings is constrained by low multiplication rates and significant phytosanitary risks. In this study, we evaluated a synthetic seed system and examined how the physicofunctional properties of sodium alginate matrices (0.8–6%) influence the ex vitro morphogenesis of axillary buds. We investigated the relationship between matrix properties and bud emergence using two distinct crosslinking agents, CaCl_2_ and Ca(OH)_2_. CaCl_2_ induced rapid gelation through the egg-box mechanism, generating densely crosslinked polymer networks with low water solubility (41–55%). These matrices may have imposed greater physical constraints on bud development reducing shoot emergence to 11.1% at low polymer concentrations and completely suppressing emergence at concentrations ≥4%. In contrast, Ca(OH)_2_ produced encapsulation matrices that supported up to 80.6% emergence, enhanced vegetative vigor, and greater biomass accumulation. Although the best encapsulated treatments showed high emergence, they did not surpass the non-encapsulated control (91.7%). Overall, sodium alginate concentrations of 0.8–1.0% provided the most favorable balance between matrix integrity and bud emergence. The results suggest that differences in matrix structural properties may influence synthetic seed performance; however, parameters such as porosity, permeability, oxygen availability, and hydration dynamics were not directly measured. These findings provide a foundation for further research on cassava synthetic seed technology. Additional studies evaluating storage longevity, transport, and field establishment are required before practical application.

## 1. Introduction

Cassava (*Manihot esculenta* Crantz) is a cornerstone of food security and socioeconomic development in tropical regions. It serves as a primary source of carbohydrates for approximately one billion people and underpins a production chain of strategic importance to Brazil, one of the world’s leading producers [1,2]. Owing to its high tolerance to low-fertility soils and water stress, cassava is widely regarded as a key crop for sustainable agriculture in the context of climate change [3]. Nevertheless, as a species predominantly propagated vegetatively, cassava faces intrinsic constraints during early establishment, including sensitivity to soil compaction, surface crust formation, and the accumulation of plant residues, all of which can compromise stand uniformity and early vigor [4,5].

The primary bottleneck limiting the acceleration of genetic improvement and the rapid dissemination of superior cultivars is the inherently low multiplication rate of conventional vegetative propagation based on stem cuttings (stakes) [6]. This system is inefficient, requiring large quantities of propagative material per unit area, exhibiting high post-harvest perishability, and showing pronounced susceptibility to desiccation. Moreover, stem cuttings serve as effective vectors for the transmission of systemic pathogens, including viruses and bacteria [7]. These limitations prolong breeding cycles, increase logistical costs, and slow varietal turnover in farmers’ fields, thereby perpetuating disease vulnerability and constraining productivity gains [8].

Recent research efforts have focused on optimizing propagation protocols through the reduction of cutting size. Evidence indicates that mini-stakes treated with protective agrochemical formulations and biostimulants can achieve agronomic performance comparable to, or even exceeding, that of standard 16-cm cuttings, while offering significant advantages in genetic parameters such as heritability and expected genetic gain [9]. This approach enables earlier multi-environment evaluations and increases the availability of propagative material during the final phases of breeding programs, potentially shortening the development cycle of new cultivars while reducing operational costs.

In parallel, advances in plant biotechnology have introduced disruptive alternatives, most notably synthetic seed technology. This approach involves the encapsulation of somatic propagules, such as axillary buds, shoot apices, or somatic embryos, within hydrated polymeric matrices that mimic the functional attributes of true botanical seeds [10]. Synthetic seeds hold considerable promise for large-scale clonal propagation, ex situ germplasm conservation, and the international exchange of plant material under high phytosanitary standards [11,12]. The feasibility of this technology has been demonstrated in several vegetatively propagated crops, including potato (*Solanum tuberosum*), in which conversion rates above 80% have been reported under optimized encapsulation conditions, as well as sweet potato (*Ipomoea batatas*), where successful emergence and plant regeneration were obtained using nodal encapsulation systems [13,14]. Synthetic seed methodologies have also been described for wild beet (*Beta vulgaris* subsp. *maritima*), highlighting their potential for germplasm conservation and clonal propagation, although standardized emergence or conversion rates were not reported in that study [15].

The economic and operational viability of synthetic seed systems depends critically on their capacity for direct field establishment (*ex vitro*), thereby eliminating the costly and time-intensive greenhouse acclimatization phase [16]. Successful protocols for *ex vitro* conversion have already been reported for crops such as sugarcane [17,18]. Success at this stage is strongly influenced by the physicochemical properties of the encapsulation matrix, which must reconcile two competing requirements: providing sufficient mechanical protection and microbiological stability during storage and transport, while enabling rapid, synchronized emergence with minimal resistance to seedling development [19].

Sodium alginate is the biopolymer most commonly used for this purpose. Extracted from brown seaweeds, this natural polysaccharide forms stable, biocompatible hydrogels through ionic crosslinking with divalent cations such as Ca²⁺ [20,21]. The structural architecture of the resulting hydrogel network—defined by alginate concentration, the type and concentration of the crosslinking agent, and gelation time—controls key properties including elastic modulus, porosity, water and gas permeability, and degradation rate [22,23]. Consequently, formulation optimization is essential to overcome the inherent trade-off between mechanical strength and physiological permeability, a central challenge in the development of effective synthetic seeds [24].

Several strategies have been proposed to modulate these properties, including the development of polymer composites. For example, the incorporation of starch into alginate matrices can function as a filler, viscosity modifier, or slow-release carbon source [25]. Similarly, the choice of crosslinking agent—calcium chloride (CaCl_2_) or calcium hydroxide (Ca(OH)_2_)—affects gelation kinetics, network homogeneity, and calcium ion release, with direct implications for the physiological performance of encapsulated propagules [26,27]. Furthermore, the incorporation of bioactive compounds, such as plant growth regulators or rooting agents, can transform the capsule into an intelligent delivery system, enhancing seedling vigor and establishment success [28].

The objective of this study was to develop and characterize polymeric films based on sodium alginate, starch, and different crosslinking agents (CaCl_2_ and Ca(OH)_2_), and to apply the optimized formulation to the encapsulation of mature cassava buds. We evaluated the physicomechanical properties of the capsules and the physiological performance of the encapsulated propagules, focusing on emergence rate and early *ex vitro* vigor. Ultimately, this work aims to establish a robust technical foundation for the use of synthetic seeds as a biotechnological tool to accelerate breeding cycles and clonal propagation in this globally important crop.

## 2. Materials and methods

### 2.1 Polymeric film production and experimental design

To develop synthetic seeds, a two-phase, complementary experimental strategy was employed. Phase I focused on the production and physicochemical characterization of model polymeric films, enabling an isolated and intrinsic assessment of encapsulation matrix properties. Phase II involved the biotechnological application of these formulations for cassava bud encapsulation, followed by agronomic evaluation. This sequential approach separates the effects of matrix material properties (Phase I) from the physiological response of the propagule (Phase II), allowing the identification of key cause–effect relationships essential for system optimization.

The physicochemical characterization performed in Phase I served as a preliminary screening step to evaluate material properties relevant to encapsulation performance, including swelling capacity, structural integrity, solubility, and biodegradability. However, film-based systems differ substantially from the three-dimensional structure and biological complexity of synthetic seeds. Therefore, the results obtained in Phase I were not intended to directly predict biological performance, but rather to support treatment selection and assist in interpreting the physiological responses observed in Phase II. Consequently, relationships between film properties and seedling performance should be interpreted cautiously, considering the structural and functional differences between the two systems.

#### 2.1.1 Formulation and production of polymeric films.

Film production and physicochemical characterization were conducted using a completely randomized design (CRD). Five sodium alginate concentrations (0.8, 1.0, 2.0, 4.0, and 6.0% w/v) were evaluated in combination with two ionic crosslinking agents, calcium chloride (CaCl_2_) and calcium hydroxide [Ca(OH)_2_], both at 10% (w/v), resulting in ten encapsulation treatments.

The 10% (w/v) concentration adopted for both calcium chloride and calcium hydroxide was used as a standardized condition to allow the isolated evaluation of sodium alginate concentration effects and direct comparison between calcium sources without interference from differences in calcium availability. Previous studies have shown that calcium concentration plays an important role in capsule formation, structural integrity, and synthetic seed performance, since adequate Ca² ⁺ levels are essential for efficient alginate crosslinking and hydrogel formation [29,25]. In addition, preliminary assays conducted in the present study indicated that this concentration provided satisfactory capsule formation and handling properties.

One corn starch–based treatment (10% w/v) and one negative control were also included, resulting in a total of 12 treatments (Table 1). Each treatment was replicated four times, and each experimental unit consisted of five Petri dishes (100 × 15 mm), resulting in a total of 240 experimental units.

**Table 1:** Experimental formulations for polymeric film and synthetic seed production.

Treatment (T)	Formulation description (concentrations, % w/v)
T1	Sodium alginate 0.8% + calcium chloride 10%
T2	Sodium alginate 1.0% + calcium chloride 10%
T3	Sodium alginate 2.0% + calcium chloride 10%
T4	Sodium alginate 4.0% + calcium chloride 10%
T5	Sodium alginate 6.0% + calcium chloride 10%
T6	Sodium alginate 0.8% + calcium hydroxide 10%
T7	Sodium alginate 1.0% + calcium hydroxide 10%
T8	Sodium alginate 2.0% + calcium hydroxide 10%
T9	Sodium alginate 4.0% + calcium hydroxide 10%
T10	Sodium alginate 6.0% + calcium hydroxide 10%
T11	Corn starch (Maizena®) 10%
T12	Control (non-encapsulated bud)

For each replication, 10 mL of the sodium alginate solution at the designated concentration were uniformly dispensed onto the bottom of sterile Petri dishes using a precision micropipette. Gelation was initiated by the slow and uniform addition of 10 mL of the crosslinking solution (CaCl_2_ or Ca(OH)_2_ at 10%). The dishes were immediately placed on an orbital shaker and agitated at 90 rpm for 5 minutes to ensure homogeneous ionic diffusion and the formation of hydrogels with uniform thickness, following established alginate crosslinking principles [30]. The resulting gels were washed three times with ultrapure distilled water (Milli-Q®) to remove excess ions and unreacted polymer. Subsequently, the gels were dehydrated in a forced-air oven at 35 ± 1 °C for 16 hours. Finally, the films were conditioned in a Biochemical Oxygen Demand (B.O.D) chamber at 27 ± 1 °C and 56 ± 5% relative humidity for 72 hours to equilibrate residual moisture and stabilize mechanical properties prior to analysis, in accordance with standardized biomaterials protocols [31].

#### 2.1.2 Physicochemical characterization of polymeric films.

Film characterization was performed to predict the functional performance of the polymeric matrices as encapsulation materials. Only sodium alginate films crosslinked with CaCl_2_ were subjected to physicochemical characterization. Films prepared with Ca(OH)_2_ did not form stable, self-supporting structures under the experimental conditions and could not be removed intact from the casting surface. Consequently, these films were not suitable for physicochemical characterization. The evaluated parameters, along with their rationale and analytical procedures, are summarized in Table 2.

**Table 2:** Parameters and analytical methods used for polymeric film characterization.

Variable	Abbreviation/ unit	Analytical procedure
Visual appearance	—	Qualitative assessment of homogeneity, transparency, presence of defects (bubbles, cracks), flexibility upon manual bending to 180°, and integrity during handling.
Moisture content	MC (%)	Indicates free water content after conditioning, influencing film stability and initial hydration rate. Discs (4 cm²) were dried at 105 ± 2 °C until constant mass (≈24 h).
Swelling index	SI (%)	Measures the hydrogel’s capacity to absorb water and swell, a critical property for water availability to the encapsulated bud in soil. Samples (2.5 × 2.5 cm) were immersed in 50 mL distilled water at 25 °C for 40 min. After surface drying, final mass was recorded.
Biodegradability in substrate	BD (%)	Evaluates matrix degradation rate in soil, defining the time window of physical protection for the propagule. Dehydrated films (4 cm²) were placed in nylon mesh bags and buried at 2 cm depth in moist substrate (70% field capacity) at 27 °C for 15 days.
Water solubility	WS (%)	Quantifies the soluble fraction of the matrix, reflecting crosslinked polymer network stability in aqueous media. Dry samples (2.7 cm²) were immersed in 50 mL distilled water under orbital shaking (130 rpm) at 25 °C for 24 h. The insoluble residue was filtered (0.45 µm), dried at 105 °C, and weighed.

### 2.2 Production and evaluation of cassava synthetic seeds

#### 2.2.1 Plant material and buds’ preparation.

Mature axillary buds from the cassava cultivar BRS Novo Horizonte were used due to their early maturity, high yield potential, and broad environmental adaptation, making this cultivar a relevant commercial genotype for the present study. Buds were collected from the middle third of lignified stems of 10–12-month-old mother plants grown under standardized conditions at the Embrapa Experimental Station. This stem region is recognized for harboring buds with greater physiological vigor and higher reserve accumulation. Stems were sectioned using a precision circular saw to obtain uninodal segments with a standardized length of 2.5 ± 0.1 cm. After cutting, the explants were disinfected by immersion in a 0.2% (v/v) quaternary ammonium (benzalkonium) solution for 10 minutes, followed by three rinses with distilled water. The explants were then surface-dried at room temperature, protected from direct airflow, for 10 min to prevent excessive bud dehydration.

The biological assay was conducted in a greenhouse using a randomized complete block design (RCBD). The blocks were established according to the spatial position of the experimental units on the greenhouse benches to account for environmental heterogeneity within the greenhouse, particularly potential differences in light distribution and microclimatic conditions associated with bench location (e.g., edge versus center positions and proximity to the side walls). The 12 treatments described in Table 1 were randomly allocated within each of the four blocks.

Each experimental unit consisted of a 4-L plastic pot containing four synthetic seeds, totaling 48 pots (12 treatments × 4 blocks) and 192 synthetic seeds. The pots were filled with a substrate composed of washed fine sand and vermiculite (3:1, v/v). Plants were maintained in a greenhouse under temperatures ranging from 25 to 27 °C, an estimated relative humidity of 60–70%, and natural photoperiod conditions. Irrigation was supplied by an automated micro-sprinkler system operating in four daily cycles of 5 minutes each, providing a standardized irrigation regime for all experimental units.

#### 2.2.2 Synthetic seed production protocol.

Encapsulation was performed using the external ionic gelation method. Sodium alginate solutions were prepared in distilled water at the concentrations shown in Table 1 under magnetic stirring until complete dissolution and deaeration. Crosslinking solutions of CaCl_2_ and Ca(OH)_2_ (10% w/v) were prepared similarly. The starch gel (T11) was prepared by gelatinization at 85 °C and subsequently cooled to 25 °C.

For encapsulation, buds from treatments T1–T10 were immersed in the alginate solution for 3 minutes to ensure complete coating and then individually transferred to the crosslinking bath (CaCl_2_ or Ca(OH)_2_) for 5 minutes, allowing the formation of calcium alginate hydrogel according to the “egg-box” model. For treatment T11, buds were immersed in starch gel for 3 minutes. The control treatment (T12) was immersed in distilled water. After encapsulation, capsules from treatments T1–T10 were washed three times with distilled water to remove excess ions and then air-dried in the shade for 1 h. Subsequently, buds from all treatments were surface-dried at room temperature, protected from direct airflow, under ambient humidity conditions typical of the experimental period. The propagules were then immediately planted in 4-L pots containing washed fine sand and vermiculite (3:1, v/v), with four synthetic seeds per pot. Pots were maintained in a greenhouse under controlled irrigation conditions.

#### 2.2.3 Agronomic traits evaluated.

The visual vigor index (StVig) was assessed using a semiquantitative scale ranging from 1 to 5 based on seedling morphological development. Scores ranged from 1 (atrophied or necrotic seedling) to 5 (vigorous seedling with at least three fully expanded leaves and a thick stem), considering leaf number, leaf expansion, stem thickness, and overall plant appearance. All evaluations were performed by a single trained evaluator using the predefined rating scale to ensure consistency in score assignment. Because all assessments were conducted by the same evaluator, inter-rater reliability was not assessed.

Only successfully established seedlings were included in the morphophysiological evaluations. Biomass measurements were performed using a standardized number of randomly selected seedlings per treatment to ensure comparability among treatments despite differences in emergence percentage (Table 3).

**Table 3:** Morphological traits measured in cassava seedlings derived from synthetic seeds.

Trait	Abbreviation	Unit	Description/ evaluation criterion
Establishment rate	Stand	%	Percentage of buds producing shoots ≥ 1 cm in length per experimental unit
Stem diameter	SD	cm	Stem diameter at the base of the plant, measured using a digital caliper (±0.01 mm).
Visual vigor index	StVig	1–5	Scale: 1 (atrophied/necrotic seedling); 3 (moderate growth, 1–2 leaves); 5 (vigorous seedling, ≥ 3 fully expanded leaves, thick stem).
Plant height	PH	cm	Distance from the collar to the apex of the main shoot.
Shoot fresh weight	SFW	g	Total fresh shoot biomass per seedling.
Shoot dry weight	SDW	g	SFW dried in a forced-air oven at 60 ± 2 °C until constant mass.
Root fresh weight	RFW	g	Total fresh root biomass per pot, carefully washed.
Root dry weight	RDW	g	RFW dried in an oven at 60 ± 2 °C until constant mass.

### 2.3 Data analysis

All analyses were performed in the R statistical environment [32]. For both Phase I (films, CRD) and Phase II (bioassay, RCBD), assumptions of analysis of variance (ANOVA), including homoscedasticity (Levene’s test) and normality of residuals (Shapiro–Wilk test), were verified (S1 Table). For each response variable, an ANOVA was conducted, and when a significant treatment effect was detected (p ≤ 0.05), treatment means were grouped using the Scott–Knott clustering test (α = 0.05). Statistical analyses were conducted using the ScottKnott package [33], and graphical outputs were generated using the ggplot2 package [34]. No data transformations were applied to Stand (%) or StVig because residual diagnostics indicated that ANOVA assumptions were satisfactorily met for both variables. Only treatments that produced sufficiently developed seedlings were included in the growth-related morphophysiological analyses.

## 3. Results

### 3.1 Physicochemical and functional characterization of CaCl_2_-crosslinked alginate matrices

**Fig 1:**
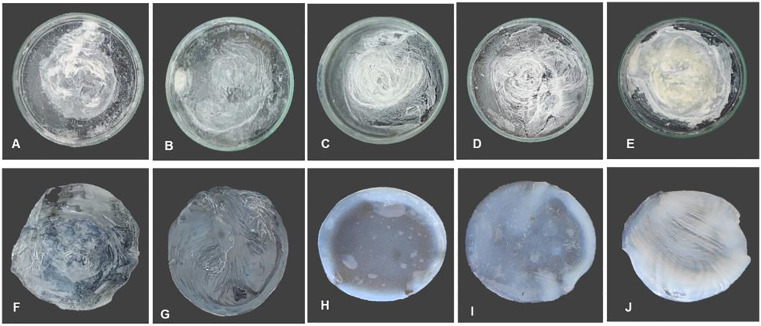
Visual appearance and structural characteristics of sodium alginate films produced at different polymer concentrations and crosslinked with either calcium hydroxide [Ca(OH)_2_] or calcium chloride (CaCl_2_). Treatments A–E correspond to sodium alginate concentrations of 0.8%, 1.0%, 2.0%, 4.0%, and 6.0%, respectively, crosslinked with 10% Ca(OH)_2_. Treatments F–J correspond to sodium alginate concentrations of 0.8%, 1.0%, 2.0%, 4.0%, and 6.0%, respectively, crosslinked with 10% CaCl_2_.

The formation and performance of polymeric encapsulation films were strongly influenced by the interaction between biopolymer concentration and crosslinking efficiency. In this study, sodium alginate films at concentrations of 0.8%, 1%, 2%, 4%, and 6% were crosslinked with CaCl_2_. These films showed satisfactory visual quality, characterized by continuous structure, surface homogeneity, adequate flexibility during handling, and the absence of major defects such as cracks or bubbles. In contrast, formulations prepared with Ca(OH)_2_ did not form stable, self-supporting films. In contrast, formulations prepared with Ca(OH)_2_ did not form stable, self-supporting films. Instead, they were opaque, brittle, and strongly adhered to the casting surface, preventing their removal intact and precluding physicochemical characterization (Fig 1). Consequently, all subsequent physicochemical analyses were performed exclusively on CaCl_2_-crosslinked matrices, which exhibited adequate structural integrity, surface uniformity, and ease of handling.

#### 3.1.1 Film moisture content.

**Fig 2:**
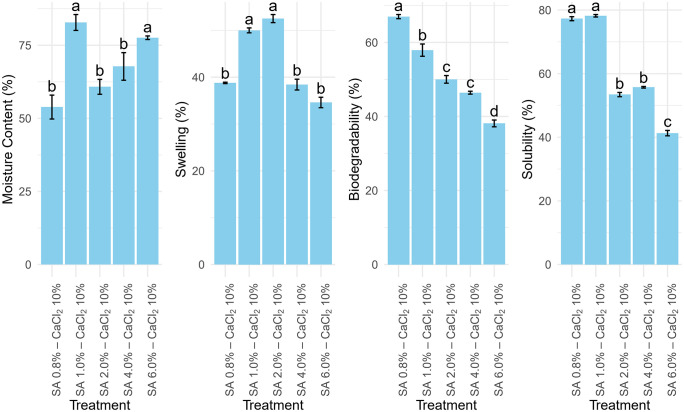
Effect of sodium alginate concentration on moisture content, swelling index, biodegradability, and solubility of CaCl_2_–crosslinked films. Means followed by the same letter do not differ statistically according to the Scott–Knott grouping test at a 5% probability level. SA = Sodium alginate; CaCl_2_ = Calcium chloride.

Film moisture content, an important parameter affecting matrix permeability and degradation kinetics, varied significantly among alginate concentrations (p ≤ 0.001; Table 4). The highest moisture content was observed in films containing 1% alginate (82.8%), followed by 6% (77.58%) and 4% (67.76%). In contrast, films prepared with 2% and 0.8% alginate showed lower moisture contents, reaching 60.78% and 53.86%, respectively (Fig 2).

**Table 4:** Analysis of variance (ANOVA) for physical and functional parameters of films developed for synthetic cassava seeds from CaCl_2_-crosslinked alginate matrices.

Source of variation	Degrees of freedom	Mean squares			
		Moisture content	Swelling index	Biodegradability in substrate	Water solubility
Sodium alginate	4	701.75***	311.10***	608.87***	1292.67***
Residual	20	86.78	26.01	37.29	13.52
Coefficient of variation (%)		29.62	55.31	19.82	11.73

*** p ≤ 0.001 (F test).

Water retention in alginate hydrogels is associated with the availability of hydrophilic groups and the physicochemical organization of the polymeric matrix [35]. The non-linear relationship observed between alginate concentration and moisture content suggests a balance between polymer hydration and network organization. Lower alginate concentrations may reduce the availability of hydrophilic interaction sites, whereas higher concentrations may promote the formation of denser polymeric arrangements that limit water retention within the matrix.

#### 3.1.2 Swelling index.

Swelling capacity, which reflects matrix hydration and expansion in an aqueous environment, also differed significantly among treatments (p ≤ 0.001; Table 4). Films containing intermediate alginate concentrations (1% and 2%) exhibited the highest swelling indices, reaching 50.0% and 52.52%, respectively. In contrast, films prepared with 0.8%, 4%, and 6% alginate showed lower swelling values of 38.78%, 38.42%, and 34.60%, respectively (Fig 2).

These results suggest that intermediate alginate concentrations may promote greater hydration of the polymeric matrix. Because permeability was not directly measured, differences in matrix permeability are proposed only as a possible explanation for the observed behavior. In contrast, very low or very high polymer concentrations may reduce hydration capacity, either because of the limited availability of hydrophilic functional groups (0.8%) or the formation of denser polymeric networks at higher concentrations (4% and 6%).

#### 3.1.3 Biodegradability.

Biodegradability, evaluated through mass loss under biologically active conditions, was strongly influenced by alginate concentration (p ≤ 0.001). Higher degradation rates were observed in less concentrated films, with values of 66.98%, 57.92%, and 50.02% for 0.8%, 1%, and 2% alginate, respectively. In contrast, films containing 4% and 6% alginate were less susceptible to degradation, showing biodegradability values of 46.42% and 38.12%, respectively (Fig 2).

These results suggest that less concentrated films are more prone to degradation, which may facilitate matrix rupture and bud emergence. Conversely, higher alginate concentrations produced more stable matrices that may restrict seedling emergence, helping to explain the low or absent emergence observed in the 4% and 6% formulations.

#### 3.1.4 Water solubility.

Water solubility is a critical functional property of the encapsulating matrix because it directly influences bud release and exposure to the emergence environment. Analysis of variance revealed a highly significant effect of alginate concentration on solubility (p ≤ 0.001; CV = 11.73%; Table 4). Films formulated with 1% and 0.8% alginate showed the highest solubility values, reaching 78.2% and 77.28%, respectively. In contrast, solubility decreased markedly at higher alginate concentrations, with values of 53.42%, 55.72%, and 41.3% for 2%, 4%, and 6% alginate, respectively (Fig 2).

At very low polymer concentrations, the formation of a continuous three-dimensional network may be impaired, causing the films to swell and fragment into partially insoluble residues, thereby reducing measured solubility. Thus, extremely diluted films may be structurally fragile while still showing incomplete dissolution. In contrast, the 1% alginate formulation produced a sufficiently cohesive matrix that promoted more complete solubilization.

Overall, the 1% sodium alginate formulation crosslinked with CaCl_2_ showed the most balanced physicochemical performance among the evaluated treatments, combining high moisture retention, elevated swelling capacity, adequate biodegradability, and high water solubility. This formulation showed the most balanced physicochemical performance among the evaluated treatments under the conditions of this study.

### 3.2 Effects of encapsulation formulations on emergence and early agronomic performance

**Fig 3:**
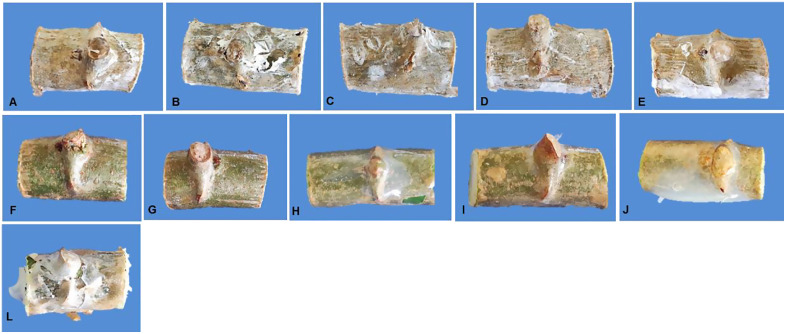
Mature cassava axillary buds encapsulated in sodium alginate matrices at concentrations of 0.8%, 1.0%, 2.0%, 4.0%, and 6.0% used for synthetic seed production. Treatments A–E correspond to sodium alginate encapsulated buds crosslinked with 10% calcium hydroxide [Ca(OH)_2_], whereas treatments F–J correspond to buds crosslinked with 10% calcium chloride (CaCl_2_). Treatment L corresponds to encapsulation using 10% corn starch. Images illustrate the effect of encapsulation matrix composition on the morphology and integrity of cassava synthetic seeds.

The effectiveness of synthetic seed technology depends on the ability of the hydrogel matrix to protect the propagule while maintaining its physiological viability. Analysis of variance revealed a highly significant effect (p < 0.001) of encapsulation formulation on all evaluated morphophysiological traits (Tables 5 and 6), indicating that matrix composition directly influenced performance from emergence to early seedling establishment. The encapsulation of cassava buds and the structural integrity of the resulting synthetic seeds are shown in Fig 3.

**Table 5:** Analysis of variance (ANOVA) for seedling emergence of cassava synthetic seeds under different encapsulation formulations.

Source of variation	Degrees of freedom	Mean Square
Sodium alginate	11	4104.10***
Block	3	391.7
Residual	30	263.8
Coefficient of variation (%)		43.56

*** p ≤ 0.001 (F test).

**Table 6:** Analysis of variance (ANOVA) for morphological traits of cassava seedlings derived from synthetic seeds under different encapsulation formulations. Only treatments with measurable seedling development were included in the analysis.

Source	DF	PH	SD	Vigor	RFW	RDW	SFW	SDW	DQI
Sodium alginate	9	0.28**	0.94***	0.86*	2.40**	0.11*	0.08***	0.002***	0.001***
Block	3	0.02^ns^	0.59*	0.14^ns^	1.32^ns^	0.05^ns^	0.02*	0.0002*	0.0002^ns^
Residuals	24	0.06	0.19	0.28	0.31	0.01	0.02	0.0002	0.0001
CV (%)	—	11.96	15.01	17.33	32.59	33.90	36.58	27.47	26.70

CV = Coefficient of variation; ns = not significant; * p < 0.05; ** p < 0.01; *** p < 0.001 (F test). PH = plant height; SD = stem diameter; SFW = shoot fresh weight; SDW = shoot dry weight; RFW = root fresh weight; RDW = root dry weight; DQI = Dickson quality index.

**Fig 4:**
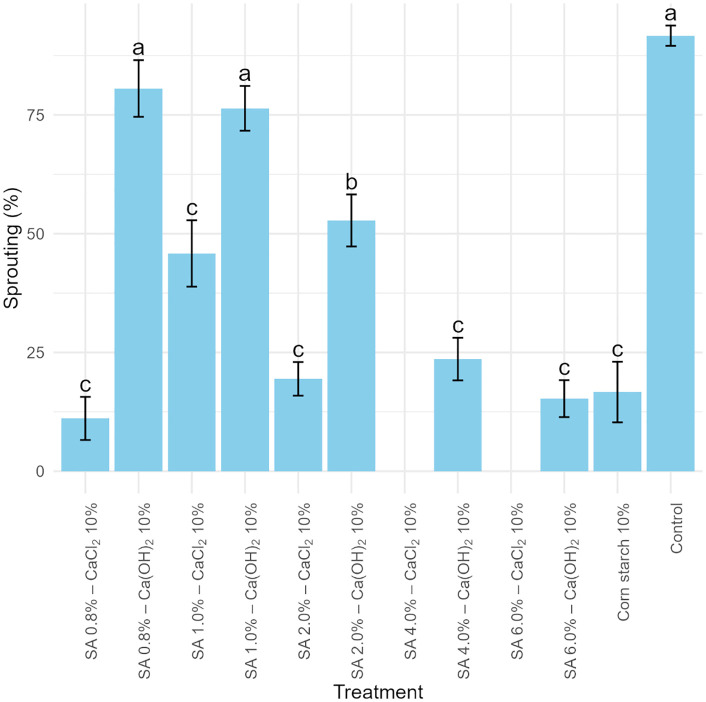
Effects of sodium alginate concentration and crosslinking agents (CaCl_2_ and Ca(OH)_2_) on bud emergence of cassava synthetic seeds. Means followed by the same letter do not differ statistically according to the Scott-Knott grouping test at 5% probability level. Error bars represent the standard error of the mean. SA = sodium alginate; CaCl_2_ = calcium chloride; Ca(OH)_2_ = calcium hydroxide.

At 20 days after planting, emergence was strongly influenced by both alginate concentration and crosslinking agent (Fig 4 and Table 5). The non-encapsulated control showed the highest emergence rate (91.7%). Among the synthetic seeds, formulations crosslinked with Ca(OH)_2_ at low alginate concentrations performed best, with SA 0.8% + Ca(OH)_2_ and SA 1.0% + Ca(OH)_2_ reaching emergence rates of 80.6% and 76.4%, respectively.

In contrast, CaCl_2_-crosslinked formulations markedly reduced emergence, with SA 0.8% + CaCl_2_ showing the lowest value (11.1%). Alginate concentrations ≥ 4% completely suppressed emergence regardless of the crosslinking agent. Treatments T4 and T5 (4% and 6% alginate + CaCl_2_) showed complete absence of emergence, representing a biologically relevant response associated with the formation of highly rigid and poorly permeable matrices. These treatments were therefore retained in the emergence dataset with zero values. However, because they did not produce measurable seedlings, they were excluded from subsequent analyses of post-emergence agronomic traits.

**Fig 5:**
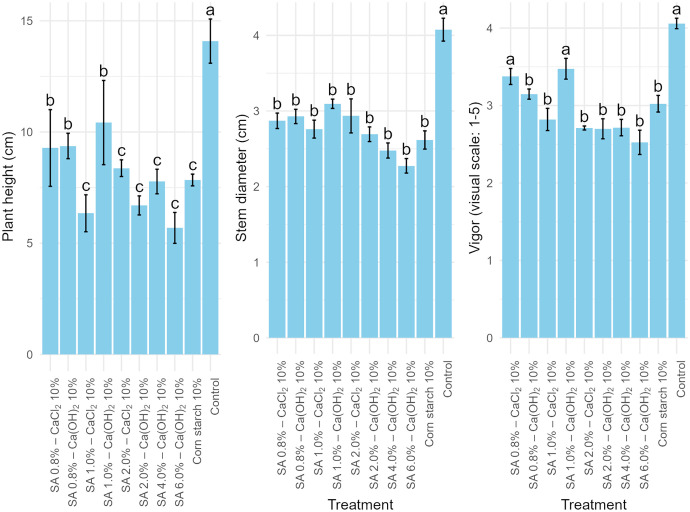
Effects of encapsulation formulations on bud emergence (plant height and stem diameter), and vigor of mature cassava buds. Means followed by the same letter do not differ significantly and belong to the same group according to the Scott–Knott grouping test at the 5% probability level. SA = Sodium alginate; CaCl_2_ = Calcium chloride; Ca(OH)_2_ = Calcium hydroxide.

Seedling vigor, which integrates emergence uniformity and developmental rate, followed a similar trend (Fig 5). The highest vigor index was observed in the control (3.71, SFW), followed by sodium alginate 1% + Ca(OH)_2_ (2.04) and sodium alginate 0.8% + CaCl_2_ (1.62). All other formulations resulted in significantly lower vigor scores.

Post-emergence growth, assessed through plant height and stem diameter, was also strongly influenced by encapsulation treatment (Fig 5). As expected, the control treatment exhibited the greatest growth, with 14.09 cm plant height and 4.08 mm stem diameter. Among the encapsulated treatments that supported emergence, seedlings were generally smaller but structurally more robust.

**Fig 6:**
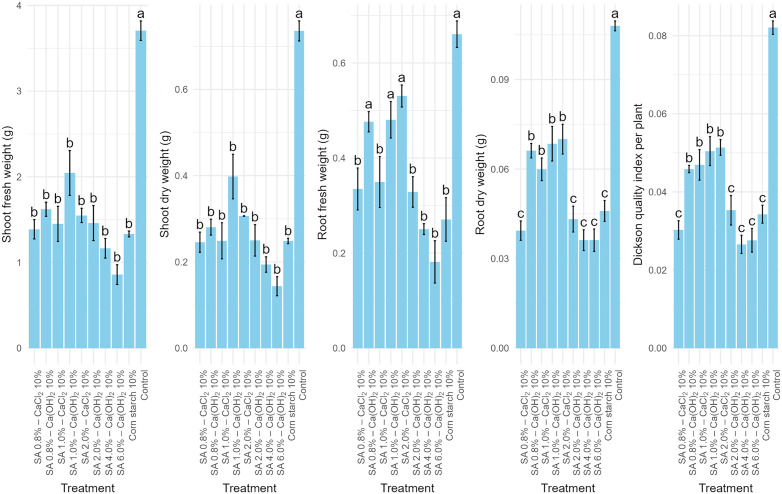
Effects of different encapsulation formulations on shoot and root biomass and Dickson Quality Index in cassava buds. Means sharing the same letter are not significantly different according to Scott-Knott grouping. Error bars represent standard error of the mean. SA = sodium alginate; CaCl_2_ = calcium chloride; Ca(OH)_2_ = calcium hydroxide.

Shoot dry weight (SDW) differed significantly among treatments (p < 0.001) (Table 6 and Fig 6). The highest value was observed in the control treatment (0.74 g), whereas encapsulated treatments showed lower biomass accumulation, ranging from 0.40 g to 0.14 g.

Biomass accumulation further highlighted the differences among formulations (Table 6 and Fig 6). The control treatment exhibited the highest shoot fresh weight (SFW; 37.06 g). Among the encapsulated treatments, low-alginate formulations crosslinked with Ca(OH)_2_ showed the highest values, particularly SA 1.0% + Ca(OH)_2_ (20.44 g) and SA 0.8% + Ca(OH)_2_ (15.84 g). In contrast, CaCl_2_-based and high-alginate formulations resulted in reductions in biomass accumulation.

Root development followed a similar trend (Fig 6). The control treatment showed the highest root fresh weight (RFW; 0.66 g), whereas the best-performing encapsulated treatments reached approximately 0.48 g. In contrast, CaCl_2_-crosslinked formulations produced the least developed root systems.

Root dry weight (RDW) also clearly differentiated the treatments. The control exhibited the highest value (0.11 g), followed by intermediate values in low-alginate formulations (0.06–0.07 g), while the lowest values were observed in high-alginate and starch-based treatments (0.04–0.05 g) (Fig 6).

The Dickson Quality Index (DQI), originally developed for forest seedlings but currently used as an integrative indicator of morphophysiological quality across different propagation systems, including vegetatively propagated plants [36], combines total biomass with the balance between root and shoot development, providing a comprehensive assessment of seedling quality and structural balance under different encapsulation formulations (Fig 6). The control treatment showed the highest DQI value (0.082). Intermediate values were observed in low-rigidity formulations, whereas the lowest values occurred in high-alginate and starch-based treatments.

Overall, excessively rigid or poorly permeable matrices not only restricted emergence but also impaired early seedling growth, resulting in lower biomass accumulation and reduced structural balance, which may compromise plant establishment under field conditions.

The most favorable emergence and early growth responses were associated with low sodium alginate concentrations (0.8–1.0%) in both crosslinking systems. However, a clear trade-off was observed between physiological performance and encapsulation integrity, which is essential for synthetic seed handling, protection, and conservation.

Formulations crosslinked with Ca(OH)_2_ (SA 0.8–1.0% + Ca(OH)_2_) promoted superior emergence, vigor, and biomass accumulation but showed lower hydrogel structural stability. In contrast, CaCl_2_-crosslinked formulations, particularly SA 0.8–1.0% + CaCl_2_, provided greater capsule integrity despite lower overall plant performance. These results suggest that although Ca(OH)_2_ favors early seedling development, CaCl_2_ plays a critical role in maintaining capsule structural integrity, highlighting the importance of encapsulation stability for the practical application of cassava synthetic seeds.

## 4. Discussion

### 4.1 Interplay between polymer matrix architecture, ionic crosslinking kinetics, and physiological performance of cassava synthetic seeds

The physiological performance of cassava synthetic seeds observed in this study appears to be strongly associated with the interaction between the physicochemical architecture of the encapsulation matrix, the kinetics of Ca² ⁺ release and diffusion, and the spatial organization of alginate chains during gelation. Together, these factors are likely to influence both the mechanical integrity of the capsule and the degree of physiological permissiveness required for shoot emergence and early seedling establishment. These findings are consistent with the classical ionic crosslinking model of alginate proposed by Grant et al. [37] and further supported by recent experimental evidence [38].

In synthetic seed systems, the encapsulating matrix functions as an artificial microenvironment that must reconcile two opposing requirements: it must provide sufficient mechanical protection during handling and manipulation while simultaneously allowing rapid hydration, gas exchange, and cellular expansion. Recent studies suggest that even subtle changes in crosslinking density and pore size distribution can lead to pronounced differences in emergence and early vigor, particularly in vegetatively propagated species [22,39]. This sensitivity was clearly evident in the present study, where relatively modest adjustments in alginate concentration and calcium source resulted in markedly different physiological responses.

Alginate remains one of the most widely used biopolymers in synthetic seed technology due to its biocompatibility, low toxicity, and ability to gel under mild conditions [40]. However, its functional performance is highly dependent on crosslinking kinetics, which are largely governed by the solubility and mobility of the calcium salt employed. Different Ca² ⁺ sources may produce polymer networks with distinct degrees of rigidity, porosity, structural heterogeneity, and temporal stability [19]. As emphasized by Sarker et al. [41], although alginate is intrinsically well suited for encapsulation, the choice of crosslinking agent is a decisive factor for biological success.

These structural characteristics may be associated with cassava physiology. Early establishment in cassava, whether from buds, sprouts, or stem cuttings, appears to benefit from permissive microenvironments characterized by adequate moisture, oxygen availability, and minimal mechanical resistance [42].This requirement is consistently reported in the synthetic seed literature, where small variations in matrix formulation significantly affect water diffusion, gas permeability, and mechanical constraints imposed on developing tissues [11,12,19,43].

In the present study, matrices formulated with lower alginate and calcium chloride concentrations were more soluble, biodegradable, and capable of swelling. These properties may have favored rapid hydration and may have reduced physical resistance to root protrusion, in agreement with reports on encapsulated systems in *Plumbago rosea* L., potato (*Solanum tuberosum* L.), and other species [44–46]. However, films produced with 0.8% alginate were excessively thin and fragile, limiting their practical applicability. Conversely, higher alginate concentrations were associated with less soluble matrices, suggesting the formation of more compact egg-box-like networks stabilized by Ca² ⁺ ions [47].

Such rigidity may have restrict water diffusion and mechanically constrained shoot emergence, a phenomenon widely reported in CaCl_2_-crosslinked encapsulation systems [25,39]. The complete absence of emergence suggests that cassava may be particularly sensitive to mechanical barriers imposed by the matrix and requires lighter, more hydrated microenvironments for meristematic tissues to rupture the encapsulating layer.

Accordingly, the formulation containing 1% alginate crosslinked with calcium chloride represented the most balanced compromise between physical integrity and physiological permissiveness within the CaCl_2_-crosslinked systems evaluated. This formulation provided sufficient mechanical stability for handling while maintaining conditions conducive to cellular elongation and bud emergence. Similar trade-offs have been identified as critical for the success of synthetic seed systems across a wide range of plant species [39,46]. In general, these results reinforce the view that synthetic seed systems should be considered dynamic rather than static structures, with matrices that evolve over time in response to hydration, microbial activity, and propagule growth. This perspective is particularly relevant for cassava, whose early developmental stages are strongly dependent on microenvironments with high water availability and low mechanical resistance [11,42].

### 4.2 Physicofunctional properties of encapsulation matrices and implications for bud emergence

Measurements of swelling, solubility, and biodegradability suggest that matrices formulated with lower alginate and Ca² ⁺ concentrations may exhibit greater structural mobility, which could facilitatate water uptake and reduce mechanical resistance to bud protrusion. This behavior mirrors observations in synthetic seeds of *Plumbago rosea*, *Solanum tuberosum*, and *Gardenia jasminoides*, where more hydratable and less compact matrices consistently promoted higher emergence rates and improved early vigor [44–46]. In contrast, higher alginate and CaCl_2_ concentrations were associated with denser, less soluble matrices and lower biodegradability, suggesting the formation of more strongly crosslinked polymer networks. While such characteristics may be advantageous for storage and transport, they were associated with lower biological performance under the conditions evaluated in this study.

Diffusion studies in alginate hydrogels have shown that increasing crosslink density substantially reduces permeability to water and oxygen, which may reduce oxygen diffusion and create microenvironments less favorable for cellular respiration. [30,48]. The absence of emergence observed in the most rigid formulations suggests that cassava buds may be particularly sensitive to initial mechanical constraints imposed by the encapsulation matrix. Comparable responses have been reported for stem cutting and sprout establishment, where soil compaction and reduced macroporosity significantly impair rooting and early growth [49].

### 4.3 Divergent effects of crosslinking agents: CaCl_2_ versus Ca(OH)_2_

A central finding of this study was the pronounced contrast between capsules crosslinked with CaCl_2_ and those formed using Ca(OH)_2_, even at identical alginate concentrations. Owing to its high solubility, CaCl_2_ promotes rapid gelation through external diffusion, resulting in the formation of a densely crosslinked surface layer commonly referred to as a “skin layer.” This structure generates a steep mechanical gradient between the capsule surface and interior, a phenomenon well documented in immersion-gelled alginate systems [38,50].

This rigid surface layer may constitute an important physical barrier to bud emergence, potentially limiting the initial elongation capacity of cassava buds. Moreover, highly crosslinked networks may restrict gas diffusion, potentially contributing to diffusional limitations within the capsule microenvironment, as reported for superstiff hydrogel systems [23].

In contrast, capsules crosslinked with Ca(OH)_2_ exhibited low cohesion, structural fragility, and rapid disintegration, particularly at lower alginate concentrations. Previous studies have reported that Ca(OH)_2_-based materials may develop more porous structures and exhibit greater calcium ion mobility, characteristics that may favor emergence and seedling physiological performance [51]. These factors may partially explain the higher DQI values observed in the present study, although the structural properties of the capsules were not directly characterized. The limited solubility of calcium hydroxide and its slower release of Ca² ⁺ ions may result in less densely crosslinked networks with fewer junction zones and greater structural heterogeneity. This configuration may favor emergence, early vigor, and root development, consistent with systems characterized by low stiffness and high deformability [39]. Nevertheless, the effects of CaCl_2_ crosslinking were not uniformly detrimental across all formulations. Intermediate alginate concentrations, particularly SA 2.0% + CaCl_2_, maintained moderate root biomass accumulation and Dickson quality index values, suggesting that moderate increases in matrix rigidity may contribute to improved structural seedling quality despite reduced emergence. Although such capsules lack the stability required for transport or long-term storage, their high physiological permissiveness highlights that biological effectiveness does not necessarily align with technological robustness.

This behavior closely reflects cassava performance under natural propagation conditions. Studies on shoot rooting consistently report superior early growth in permissive microenvironments with high water diffusion and minimal mechanical constraints. Light, well-aerated substrates combined with adequate moisture strongly enhance root initiation and sprout vigor [49]. Similar responses have been documented in other species (*Plumbago rosea* L., *Gardenia jasminoides*, *Daucus carota*, and *Solanum tuberosum*) encapsulated with CaCl_2_, where excessive matrix rigidity limits early emergence, underscoring the broad interspecific relevance of the trade-off between mechanical stability and physiological permissiveness [25,43,45,46].

### 4.4 Polymeric films as model systems for cassava bud encapsulation

Characterization of polymeric films prepared using the same formulations as the capsules provided valuable insights into the structural and functional behavior of encapsulation matrices. Films produced with higher alginate and CaCl_2_ concentrations exhibited elevated moisture retention, greater swelling capacity, low solubility, and reduced biodegradability, suggesting the formation of denser and more strongly crosslinked polymer networks with lower structural mobility [12,26].

Recent studies on biopolymeric films and hydrogels has demonstrated that matrix composition directly influences key properties such as stiffness, permeability, water absorption, and structural stability, supporting the use of two-dimensional systems as predictive models for three-dimensional encapsulation behavior [52,53]. In this context, the present results suggest that films may serve as useful screening tools for the preliminary assessment of encapsulation matrix performance. Conversely, films formulated with only 0.8% alginate were extremely thin, highly soluble, and mechanically unstable, suggesting the formation of a less cohesive polymer network. The observed correspondence between the physicofunctional properties of the films and the physiological performance of the capsules suggests that these systems may be useful for evaluating swelling behavior, mechanical resistance, and degradation dynamics. Although extrapolation from two-dimensional films to three-dimensional capsules has inherent limitations, including the absence of internal diffusion gradients and curvature effects, films nonetheless provide a useful experimental platform for formulation optimization and for evaluating the effects of polysaccharide concentration, crosslinking intensity, and additive incorporation in synthetic seed matrices [12,26,52,53].

### 4.5 Physiological sensitivity and the cost of encapsulation

Even under the most effective formulations, seedlings derived from synthetic seeds consistently performed below the non-encapsulated control, suggesting physiological cost associated with encapsulation the conditions evaluated in this study. Previous studies suggest that this effect may be associated with multiple, interconnected mechanisms, including: (i) diversion of metabolic energy toward rupture or degradation of the polymeric matrix; (ii) delayed hydration of meristematic tissues; and (iii) possible limitations in the diffusionof water, nutrients, and oxygen within the capsule microenvironment [11,12].

In cassava, this physiological cost may be particularly relevant because successful establishment depends on rapid root development and early seedling vigor. Accordingly, the main technological challenge of encapsulation does not lie in completely eliminating this cost rather than completely eliminating this physiological cost in polymer-based systems—but rather in minimizing it through the design of bioresponsive matrices capable of degrading, solubilizing, or deforming in synchrony with propagule growth. Such an approach may help reconcile early mechanical protection with physiological permissiveness, thereby supporting future developments in cassava synthetic seed technology for clonal propagation, germplasm conservation, and vegetative material logistics.

### 4.6 Fundamental dualities in synthetic seed technology

The trade-off between structural rigidity and physiological emergence observed here is not exclusive to cassava and has been reported in other encapsulated species, including *Mentha spicata* [24] and *Brassica oleracea* [54]. This cross-species recurrence suggests that the challenge of balancing mechanical stability and permeability may represent a common challenge in alginate-based encapsulation systems, although species-specific physiological responses are also likely to contribute to overall performance.

The present results support the existence of this apparent duality in matrices formulated with alginate crosslinked using either CaCl_2_ or Ca(OH)_2_. More rigid networks may provide greater technological stability but impose physical and diffusional constraints that limit emergence. Conversely, less dense and more deformable matrices may favor emergence and early vigor but are mechanically fragile. This dichotomy has been widely recognized as a central limitation of synthetic seed technology, as system performance critically depends on balancing mechanical protection with physiological permissiveness [55].

This limitation is especially pronounced in vegetatively propagated species such as cassava, whose early establishment phase is highly sensitive to physical impediments and appears to benefit from microenvironments that promote cellular expansion, high water availability, and unrestricted root growth [42,49]. Thus, the inferior performance observed in excessively rigid matrices may reflect both. structural inadequacy of the capsule, but also the species’ heightened sensitivity to conditions resembling compacted soils or environments with reduced macroporosity. Although this study focused exclusively on alginate matrices crosslinked with CaCl_2_ or Ca(OH)_2_, these formulations represent only a subset of available technological options. This limitation constrains the generalization of the findings to more complex or hybrid encapsulation systems and highlights the need for future studies exploring alternative compositions, architectures, and encapsulation strategies capable of mitigating the identified structural duality.

### 4.7 Future directions for overcoming structural duality

The duality between mechanical stability and physiological permissiveness identified in this study appears to represent of systems based solely on ionic alginate gelation [19,54]. Overcoming this constraint will likely require advanced materials engineering approaches capable of decoupling global mechanical strength from local deformability, allowing capsules to simultaneously meet technological and physiological requirements.

One promising avenue for future research is the development of time-responsive encapsulation systems with stage-dependent functionality during propagule establishment. In such systems, the capsule could initially provide a less restrictive and more permeable microenvironment to facilitate bud emergence and root protrusion, while subsequently maintaining sufficient structural integrity and water retention to support seedling establishment. Although this concept was not evaluated in the present study, it aligns with the broader objective of designing encapsulation systems that balance physiological permissiveness with technological protection. Research on such adaptive matrices remains limited in cassava synthetic seed technology and represents an important opportunity for future investigation.

Promising strategies for future investigation include: (i) interpenetrating polymer networks (IPNs) combining ionic alginate crosslinking with mild covalent crosslinking (e.g., gelatin or other biopolymers), which enhance toughness and fracture resistance without substantially compromising permeability [56]; (ii) biopolymeric nanocomposites reinforced with cellulose nanofibers, nanoclays, or related nanomaterials, enabling reductions in alginate concentration while maintaining adequate mechanical strength enabling finer control over matrix structure,/and biodegradation; and (iii) functional or “smart” synthetic seeds incorporating plant growth regulators, beneficial microorganisms, or nutrients, transforming the capsule from a passive enclosure into an active physiological modulation system with the potential to partially offset.

In addition to improvements in capsule architecture, future studies should evaluate the storage longevity and post-storage performance of encapsulated cassava propagules. Although the present study demonstrated successful ex vitro establishment of synthetic seeds produced from single-bud stem segments, the practical application of this technology is likely to depend on maintaining propagule viability during storage and transport. Consequently, investigations addressing shelf-life extension, storage conditions, and post-storage emergence will be important for translating this technology from experimental validation to practical deployment.

This study has some limitations that should be considered when interpreting the results. Although greenhouse temperature and relative humidity were monitored throughout the experiment, light intensity and the exact irrigation volume applied during each irrigation cycle were not quantified. These environmental factors may have contributed to variation in seedling establishment and early growth. Future studies incorporating these measurements may provide a more comprehensive understanding of their influence on the performance of encapsulated cassava buds.

In this context, the present study provides a conceptual and experimental foundation by defining the performance limits of classic alginate-based formulations for synthetic cassava seeds. At the same time, it identifies promising directions for the development of next-generation encapsulation systems with enhanced structural and functional sophistication, thus supporting future advances of this technology for clonal propagation, germplasm conservation, and efficient logistics of vegetative material. The successful use of highly reduced propagules consisting of single-bud stem segments further suggests the potential of this approach to contribute to increasing multiplication rates and improve the efficiency of cassava propagation systems.

## 5. Conclusion

This study demonstrates that the performance of cassava synthetic seeds is strongly influenced by the physicochemical architecture of the encapsulation matrix, which is determined by the interaction between alginate concentration and the calcium source used for crosslinking. Variations in capsule rigidity and structural integrity, which may be associated with differences in hydration dynamics and matrix organization, were accompanied by contrasting physiological responses, highlighting the importance of the encapsulation matrix as an artificial microenvironment for bud emergence and early seedling establishment. Among the CaCl_2_-crosslinked formulations, sodium alginate concentrations of 0.8–1.0% (SA 0.8% + CaCl_2_ and SA 1.0% + CaCl_2_) provided the most favorable balance between seedling emergence and capsule structural integrity, making them the most suitable formulations among those evaluated for synthetic seed production and handling. In contrast, the best agronomic performance was consistently observed in low-concentration sodium alginate formulations (0.8–1.0%) crosslinked with Ca(OH)_2_, particularly SA 0.8% + Ca(OH)_2_ and SA 1.0% + Ca(OH)_2_. These formulations promoted higher emergence rates, greater seedling vigor, and superior early biomass accumulation compared with all CaCl_2_-crosslinked and high-alginate treatments. Nevertheless, none of the encapsulated formulations surpassed the performance of the non-encapsulated control.

The results indicate that the performance of synthetic seed systems depends on achieving an appropriate balance between mechanical protection and physiological permissiveness. The findings provide a foundation for the further development of alginate-based encapsulation systems for cassava. However, additional studies evaluating storage longevity, transport, field establishment, and large-scale validation are required before practical application.

## Supporting information

S1 Table Results of the Shapiro–Wilk normality test and Levene’s test for homogeneity of variances based on model residuals for the evaluated variables. (DOCX)
