# Supplementary material for: Development and evaluation of a synthetic seed system for cassava propagation
Source: PLoS One. 2026 Aug 14;21(8):e0356235. doi: 10.1371/journal.pone.0356235 (PMC13475825; doi:10.1371/journal.pone.0356235)
Supplement: Supplementary file 1 [file pone.0356235.s001.docx]

**Table S1**. Results of the Shapiro–Wilk normality test and Levene’s test for homogeneity of variances based on model residuals for the evaluated variables.

| **Variable** | **Shapiro–Wilk (W)** | **p-value** | **Levene’s test (F)** | **p-value** |
| --- | --- | --- | --- | --- |
| Sprouting | 0.97 | 0.50 | 1.45 | 0.22 |
| PH | 0.98 | 0.77 | 2.94 | 0.01 |
| DH | 0.97 | 0.46 | 0.18 | 0.99 |
| Vigor | 0.97 | 0.33 | 0.38 | 0.93 |
| PFR | 0.98 | 0.57 | 1.11 | 0.39 |
| PSR | 0.98 | 0.68 | 1.13 | 0.38 |
| PFPA | 0.98 | 0.79 | 1.23 | 0.32 |
| PSPA | 0.99 | 0.89 | 1.37 | 0.25 |
| IQD | 0.98 | 0.72 | 1.76 | 0.12 |

Sprouting = sprouting percentage; PH = plant height; DH = hypocotyl diameter; Vigor = seedling vigor score; PFR = fresh root mass; PSR = dry root mass; PFPA = fresh shoot mass; PSPA = dry shoot mass; IQD = Dickson quality index. Plant height (PH) data were log-transformed prior to analysis to satisfy the assumption of homogeneity of variances.
